# Toward an effective use of laser-driven very high energy electrons for radiotherapy: Feasibility assessment of multi-field and intensity modulation irradiation schemes

**DOI:** 10.1038/s41598-020-74256-w

**Published:** 2020-10-14

**Authors:** Luca Labate, Daniele Palla, Daniele Panetta, Federico Avella, Federica Baffigi, Fernando Brandi, Fabio Di Martino, Lorenzo Fulgentini, Antonio Giulietti, Petra Köster, Davide Terzani, Paolo Tomassini, Claudio Traino, Leonida A. Gizzi

**Affiliations:** 1 Consiglio Nazionale delle Ricerche, Istituto Nazionale di Ottica, Pisa, Italy; 2 Consiglio Nazionale delle Ricerche, Istituto di Fisiologia Clinica, Pisa, Italy; 3 Unità Operativa di Fisica Sanitaria, Azienza Ospedaliero-Universitaria Pisana, Pisa, Italy; 4 Present Address: Lawrence Berkeley National Laboratory, LBL, Berkeley, CA USA

**Keywords:** Plasma-based accelerators, Radiotherapy

## Abstract

Radiotherapy with very high energy electrons has been investigated for a couple of decades as an effective approach to improve dose distribution compared to conventional photon-based radiotherapy, with the recent intriguing potential of high dose-rate irradiation. Its practical application to treatment has been hindered by the lack of hospital-scale accelerators. High-gradient laser-plasma accelerators (LPA) have been proposed as a possible platform, but no experiments so far have explored the feasibility of a clinical use of this concept. We show the results of an experimental study aimed at assessing dose deposition for deep seated tumours using advanced irradiation schemes with an existing LPA source. Measurements show control of localized dose deposition and modulation, suitable to target a volume at depths in the range from 5 to 10 cm with mm resolution. The dose delivered to the target was up to 1.6 Gy, delivered with few hundreds of shots, limited by secondary components of the LPA accelerator. Measurements suggest that therapeutic doses within localized volumes can already be obtained with existing LPA technology, calling for dedicated pre-clinical studies.

Laser-plasma acceleration (LPA) of relativistic electrons via the Laser Wakefield Acceleration (LWFA) mechanism^1^ has seen an amazing progress over the past decade, marked by the recent dramatic increase of the maximum electron energy^2–4^, enabled by advanced laser guiding schemes^3^ and by a deeper understanding of the underlying physics of electron injection and acceleration^5^. In fact, several injection methods have been proposed^6–8^ leading to impressive improvements of the beam quality in terms of energy spread, beam emittance and/or divergence and bunch charge; staging has also been successfully investigated^9,10^, allowing $\sim \,\mathrm {GeV}$-scale beams with “accelerator-like” quality to be designed (see Refs.^11,12^ and references therein). Stability and reproducibility of the principal beam parameters have also been steadily improving. In light of this progress, efficient production of secondary particles and/or high energy photons have become possible^13–16^, as well as the investigation of strong field QED phenomena^17,18^.

One of the key promising features of LPA sources relies in their high acceleration gradient, leading to extreme compactness, resulting, ultimately, in reduced costs, as well as in a potential for a much broader availability with respect to conventional, RF-based accelerators. In this respect, among all possible applications (see for instance^19^ and Refs. therein), exploitation of LPA sources in biology and medicine appears particularly appealing, in view of novel applications and protocols to be conceived and new devices deployed in the medical practice. In particular, the great potential of laser-based X-ray sources for advanced biological and medical imaging has been recognized and investigated (see Ref.^20^ and Refs. therein), also in combination with conventional sources^21^. For instance, the sub-micrometer size of the emitting region of laser-driven sources such as (all-optical) Thomson Scattering^22–24^ or betatron^25,26^ sources makes them ideal for potentially enabling phase-contrast imaging of biological specimens, currently mostly limited to large scale 3rd or 4th generation light sources, to be carried out in medium scale laboratories^27–29^. Moreover, the very short pulse duration of such X-ray sources, coupled to their collimation and resulting, at the same time, in a high brightness, opens perspectives for novel applications in dynamic $\mu$CT imaging^30^.

A similarly exciting, but likely more challenging, opportunity for the use of LPA sources in the medium term can be envisaged in the field of radiotherapy. In this context, laser-driven particle accelerators with “medical” quality would indeed open new perspectives for their widespread use (see Ref.^31,32^ and Refs. therein). Latest figures from the International Agency for Research on Cancer (IARC) show an annual global burden of new cancer cases estimated in around 18 million in 2018, with more than 9 million deaths in the same year, making cancer among the main causes of death^33,34^. Radiotherapy is currently recognized as a key component in the management of cancer patients, used alone or in combination with other treatments, mainly including surgery, chemotherapy, immunotherapy and hormonal therapy. Around 50% of all cancer patients generally undergo a kind of radiotherapy treatment^35^. Having in mind the increasing worldwide need for radiotherapy devices, recently stressed by the Global Task Force on Radiotherapy for Cancer Control^36^, the development of laser-driven accelerators, with their potential practical advantages, could have a major impact. From a practical viewpoint, we just mention here that a 100TW class laser system, capable of driving LPA of electrons up to hundreds of MeV, exhibits a typical footprint of $\sim 10\,{\mathrm {m}}^{2}$; this is in stark contrast to an equivalent RF LINAC. Furthermore, the electron acceleration stage consisting only of a centimeter scale device, coupled to the relative easiness of steering the laser (optical) beam, enable even more attractive features, such as the capability to serve different radiotherapy stations using a single laser driver or the compactness of radioprotection structures, required only for the small acceleration stage downstream of the laser system.

As it is well known, radiotherapy is nowadays mostly delivered using $\gamma$-ray photons; hadrontherapy is currently restricted to a limited number of installations worldwide, mainly due to cost constraints (see for instance^37^ and Refs. therein); research toward laser-driven hadrontherapy is also ongoing^38–41^. Direct use of electrons plays a relatively minor role in today’s radiation therapy, essentially due to the low penetration depth at the energy range available with current clinical level linear accelerators ($\sim$ 6–25 MeV). In fact, clinical application of electrons is currently mainly limited to the so-called intra-operatory radiation therapy (IORT)^42^, which employs electron bunches with energy up to $\sim$ 10 MeV to provide a superficial (down to a few mm in depth) dose boost to the tumor bed immediately following its surgical removal.

It was clear since the end of the nineties that the use of the so-called Very High Energy Electrons (VHEE), with an energy above $\sim$ 50–100 MeV and maximum energy up to $\sim$ 250 MeV, while being required to reach most of the deep seated tumors^43,44^, would also allow the lateral dose spread to be limited, enabling a precise dose deposition.

Over the past 2 decades, the potential of electron radiotherapy for deep-seated tumors has been investigated also in view of possible further developments of VHEE accelerators, either RF-based^44–46^ or laser-driven^47^. For instance, in^48^, the development of a treatment planning for VHEE in the range up to 200 MeV is reported, where results from Monte Carlo simulations show similar or superior dose distributions in pediatric, lung and prostate cancer as compared to clinical VMAT plans. A similar study was reported in^49^ for prostate cancer, starting with a typical LPA VHEE beam. Remarkably, LPA sources have indeed been emphasized as a unique solution for delivering a VHEE beam in a reduced footprint compared to conventional RF accelerators. From an experimental viewpoint, the first studies were aimed at characterizing LPA bunches from a dosimetric point of view^50–54^. Furthermore, testbeds for the irradiation of small animals were developed and preliminary experiments carried out^55^. On the other hand, a discrete number of studies have been carried out over the past few years, aimed at assessing the cell damage effects of laser-driven VHEE, measured for different biological endpoints. Indeed, as it is well known^56,57^, the typical duration of LPA electron bunches is several orders of magnitude smaller than that of “conventional” RF bunches used for radiotherapy. However, since the charge per bunch is comparable, this results in a much higher *peak* dose rate, raising an issue on the possible difference in the biological damage of cells. It was thus soon recognized that experimental studies in the field of ultrahigh peak dose rate radiobiology would be of a paramount importance for the translation of LPA beams to the clinical practice. Moreover, the extremely short duration of LPA bunches allows possible novel phenomena in radiobiology to be investigated, possibly gaining access to the very early phase of the damage induced on a particular cell structure by the primary ionizing particle^58^. Several works have been reported in the field over the past decade^50,59,60^.

In spite of the remarkable number of theoretical and numerical studies related to the use of VHEE beams for radiotherapy, and of experimental dosimetric and radiobiological studies, to our knowledge no experiment has been reported so far aimed at assessing the feasibility of advanced irradiation methodologies typical of current radiotherapy practice. In fact, for all non-superficial tumors, the outcome of any radiotherapy treatment is strongly related to the ability to deliver the highest possible damage to cancer cells while preserving the integrity of nearby tissues, especially the organs at risk (OAR). 3D conformal radiation therapy (3DCRT) with external MV photon beams, subsequently replaced by intensity modulated radiation therapy (IMRT), is being used since decades. More recent approaches include volumetric modulated arc therapy (VMAT) and helical tomotherapy. Due to the exponential attenuation of photons through the patient’s body, irradiation from different directions (multi-field irradiation, MF) and suitable shape and intensity modulation (IM) are required to limit the dose to non-target tissue while keeping the desired effect to the planned target volume (PTV). As shown by the treatment planning studies related to the use of VHEE cited above, this is also true for irradiation with electron beams. Preliminary experimental investigations of these irradiation schemes with real VHEE beams are thus of a crucial importance. This is true, in particular, in view of the unique features of typical LWFA bunches, like the higher divergence or the broad energy spectrum of basic high bunch charge LPA configurations.

In this paper, we demonstrate that VHEE generated by laser-plasma accelerators can be used to control dose deposition, using similar stereotactic configurations and with intensity modulation techniques, as with conventional high energy X-ray beams, providing a suitable platform for in vivo studies and paving the way to the practical implementation of VHEE radiotherapy.

**Figure 1:**
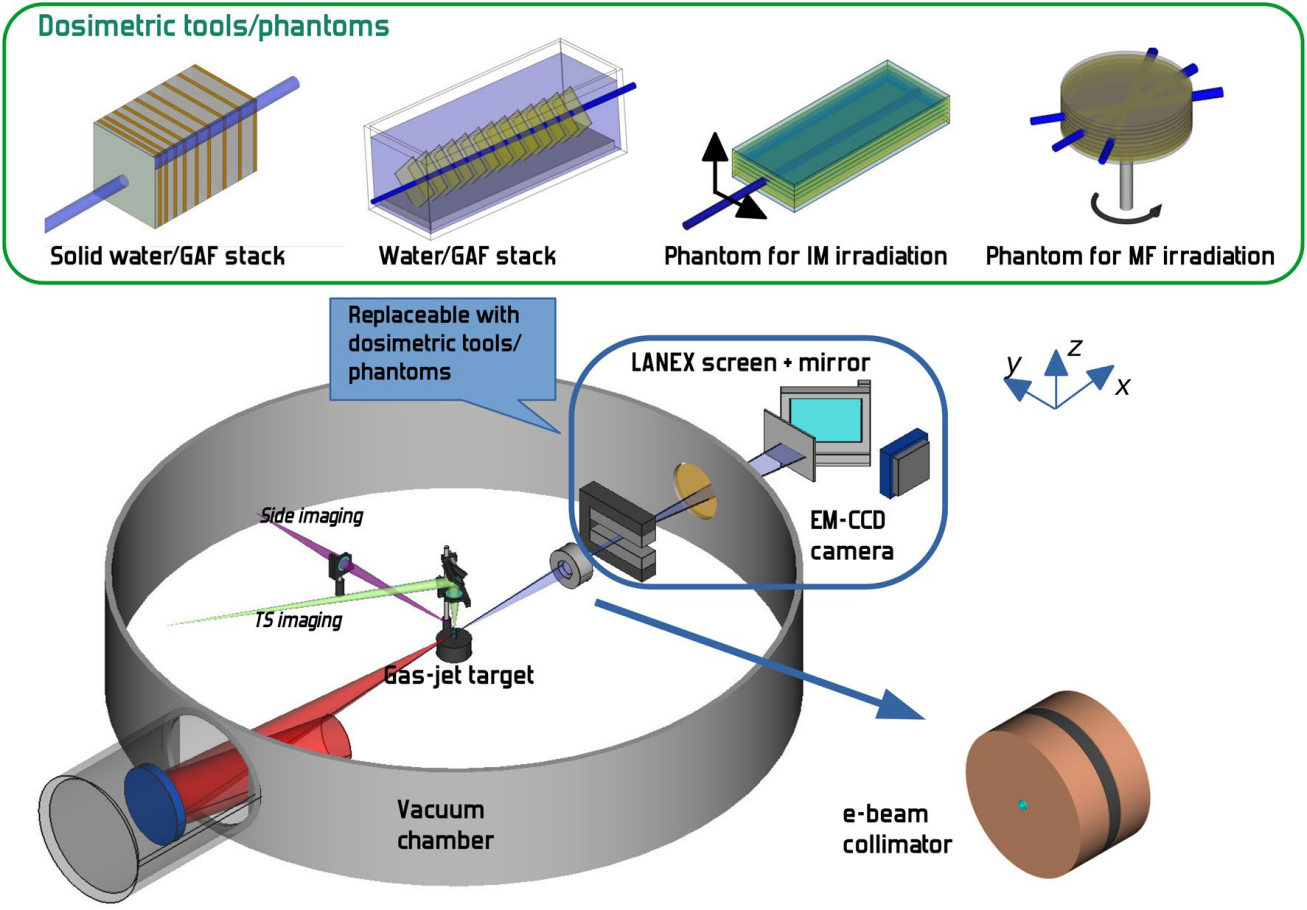
Rendering of the main phantoms and of the setup used for the experiment. The four phantom/gafchromic film detector assemblies used in the measurements are shown in the green frame. The vacuum chamber hosts the main LPA components, namely the laser focusing Off-Axis Parabola and the gas-jet plasma target, along with LPA monitoring diagnostics including the Thomson Scattering imaging and side imaging. A 2 mm aperture electron beam collimator was placed $\sim$ 38 cm downstream of the gas-jet nozzle. The electron beam diagnostic set, including the magnetic dipole, the LANEX screen and the camera, was taken out of the beam path when irradiation of phantoms for dosimetry, multi-field and intensity modulation experiments took place.

## Results

### Electron acceleration and bunch dosimetry

The experimental work reported here was carried out using the 220 TW beamline of the TiSa laser system at the Intense Laser Irradiation Laboratory at CNR-INO (Pisa, Italy)^61^. Specifically, for this experiment laser pulses with $\gtrsim 3\,\mathrm {J}$ energy in the focal spot and $30\,\mathrm {fs}$ duration were employed, focused to an intensity of $6\times 10^{18}\,{\mathrm{W}}/{\mathrm{cm}}^2$ ($a_0\simeq 1.7$) on a gas-jet target consisting of a He–N$_2$ mixture. Figure 1 shows a sketch of the experimental setup. We provide in the “Methods” section more details on laser focusing, target, plasma and electron bunch diagnostics. The electron bunches produced exhibited a pretty good collimation [measured divergence $\sim \,$14 mrad (FWHM)]. Throughout our experiment we used a passive collimator, placed at about 38 cm downstream of the gas-jet to further reduce the electron beam size on the sample. The structure of the collimator was designed ad hoc, using Monte Carlo simulations, so as to minimize dose contamination from *Bremsstrahlung *photons (see “Methods” section and Supplementary Materials).

For the irradiation experiments described below, an LPA operating regime was used providing electron bunches with most of the charge in an energy range from $\sim 50$ to $\sim 250\,\mathrm {MeV}$. As anticipated above, this range corresponds to the ideal range of VHEE energy expected to be suitable for the treatment of the great majority of deep tumors. We refer to the Supplementary Materials for a discussion of the acceleration process taking place in our experimental conditions and of the spectral features of our bunches. Here we mention that a rather broad spectrum was aimed at, covering essentially the whole VHEE range of interest for radiotherapy (in our case the spectrum went up to $\sim$ 250 MeV). In particular, our spectrum was relatively flat in the region from $\sim 100\,\mathrm {MeV}$ upward, resulting in a greater dose-per-pulse with respect to previous experiments (see for instance^62^ and Refs. therein). This leads to a non-ideal longitudinal dose deposition profile, according to the consolidated studies and treatments, and will eventually require optimization. We will further elaborate on this point in the Discussion.

As for the underlying acceleration mechanism in our conditions, Particle-In-Cell (PIC) simulations were intensively employed to get insights on it. We refer to the “Methods” section and Supplementary Materials for details about this point. Here we mention that ionization injection^63^ from nitrogen electrons was found to play the main role in this regime. It is worth observing that shot-to-shot stability of all the bunch parameters is of a crucial concern for real clinical applications; in our case we observed both spectrum and charge to be rather stable when averaged over 10 laser shots. We will further elucidate this point in the Discussion.

**Figure 2:**
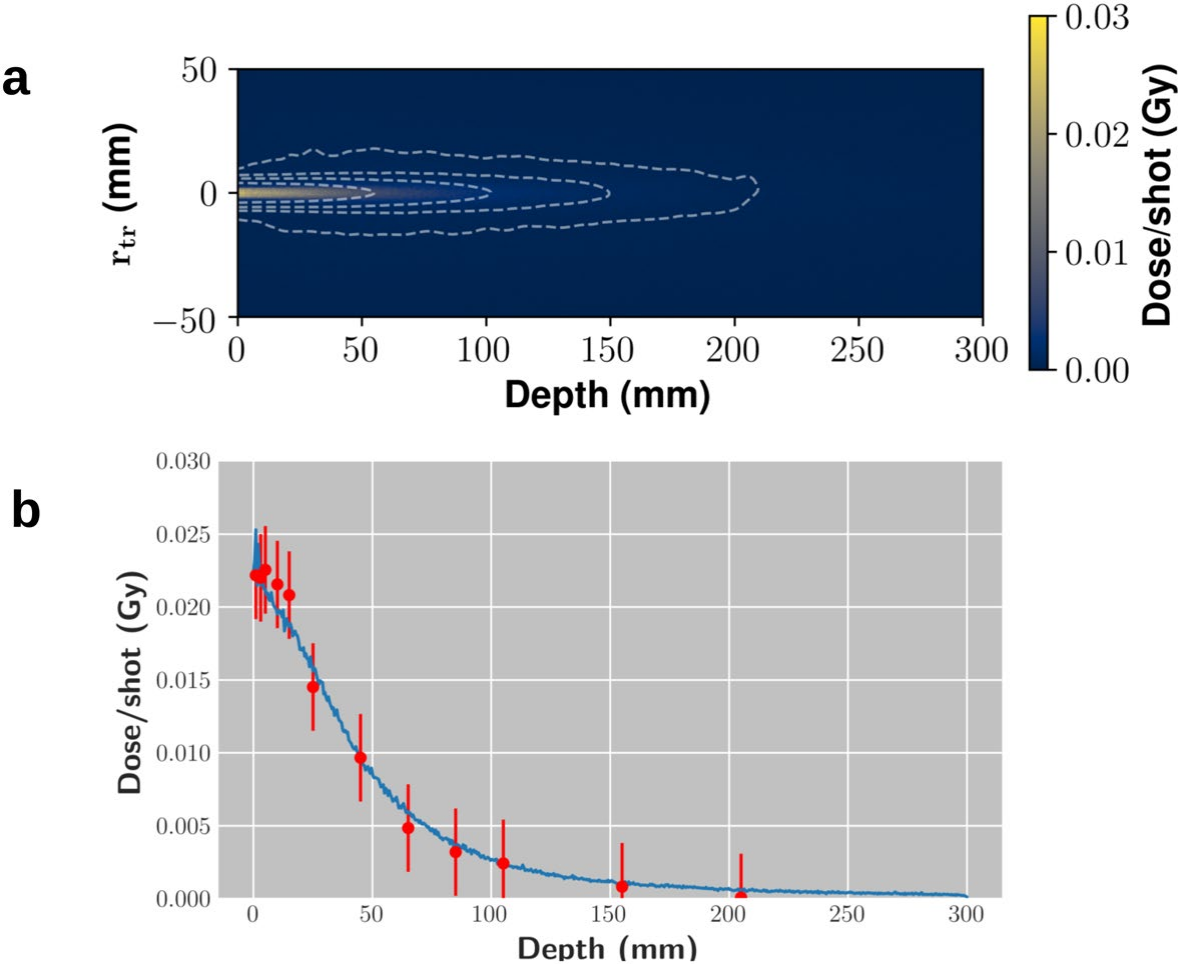
**a** 2D slice of the dose distribution in a water phantom, as obtained by Monte Carlo simulations. Isodose countours are shown, for dose levels 0.0005, 0.001, 0.002 and $0.005\,\mathrm {Gy}$. **b** Percentage Dose Depth (PDD) curves as retrieved from the experimental measurements (points) and as obtained by Monte Carlo simulations (continuous line).

The dosimetric features of the bunch were investigated using either a stack of solid water plates or a water filled tank. In both cases, gafchromic films (model EBT3) were used as a detector (see “Methods” section for details), and the deposited dose was tracked up to a depth of approximately 20 cm. In Fig. 2 (lower plot) the experimental dose on axis at different depths in the solid water phantom, corresponding to the so-called Percentage Dose Depth (PDD) curve, is shown. These data were compared to Monte Carlo simulations, carried out using the GEANT4 library toolkit, in order to both retrieve the bunch charge and predict the dose deposition pattern in the multi-field and intensity modulation experiments discussed below. The experimental spectrum and divergence were used in these simulations for the primary electrons. The bunch charge was estimated to be of about 120 pC/shot, in a rather good agreement with PIC simulations. The PDD curve as resulting from the GEANT4 simulations is shown in the plot of Fig. 2, along with the experimental points. We observe that in our experimental configurations the useful spectrometer range had a lower energy threshold of about 50 MeV. In the GEANT4 simulations, no electrons were considered below this threshold; this might in principle result in a degree of inaccuracy for the dose deposition pattern at small depths. Finally, we show in the top plot of Fig. 2 a 2D map of the dose deposition pattern inside a solid water phantom, as predicted by the Monte Carlo simulations, obtained from a cut along a plane parallel to the electron beam propagation direction. As it is clear, the dose pattern of the initial beamlet, which exhibits a transverse size of a few mm at the entrance, remains confined up until a depth of 80 mm. As already observed by earlier numerical studies^43^ this is a remarkable feature of VHEE beams, in sharp contrast to lower energy electron beams, ultimately making them suitable for radiotherapy of deep tumors.

**Figure 3:**
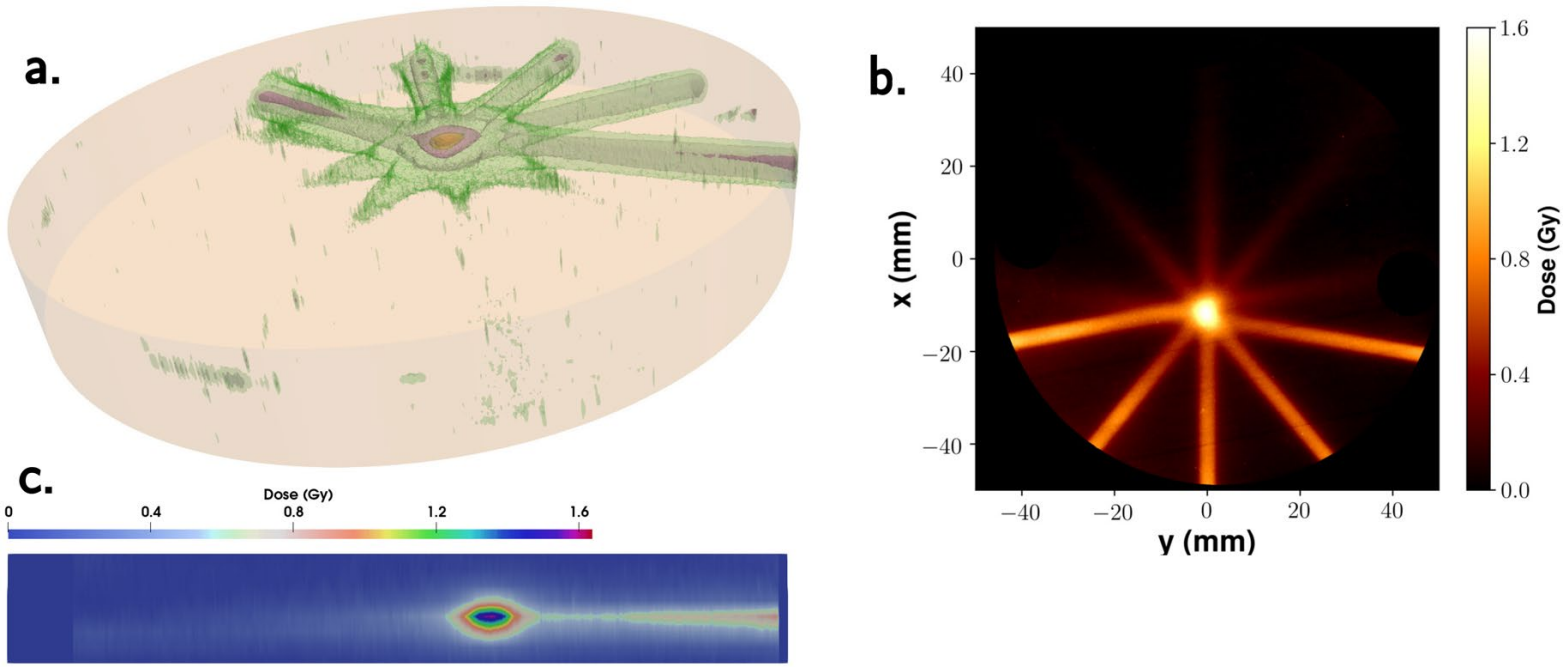
Experimental dose distribution retrieved from experimental data in the MF irradiation case. **a** Isodose surfaces: green: 0.2 Gy, grey: 0.4 Gy, pink: 0.8 Gy, yellow: 1.2 Gy, red: 1.4 Gy As a visual aid, the light orange cylinder stands for the phantom. **b** 2D slice along the *z* direction (see Fig. 1 for the coordinate system used), taken at the (*z* constant) position of the e-beam. **c** 2D slice along the *y* direction, taken at the (*y* constant) position corresponding to the center of the central field.

### Advanced RT methodologies with VHEE

We now present the results of experiments aimed at mimicking the complex irradiation schemes and methodologies currently employed in radiotherapy. In particular, two basic building blocks can be identified, namely the so-called multi-field irradiation (MF) and the intensity modulation (IM). We preliminarily observe that current radiotherapy protocols are based on the delivery of “elementary” dose patterns by means of the so-called pencil beamlets, with a typical transverse size of the order of a few mm up to 10 mm^48^, whose combination is capable of covering typical areas up to $\sim$ 10 cm wide. Generation of these pencil beams out of rather large and uncollimated *Bremsstrahlung* photon sources is achieved through a multi-leaf collimator^64^. In our experiment we set the transverse size of the electron beamlet so as to demonstrate the possibility to tailor the dose deposition with the required precision, and over the typical volumes encountered in the clinical practice.

Figure 1 (insets MF and IM) shows the phantoms used for the experiment. In the case of MF irradiation, the phantom consisted of thin (2 mm thickness) polycarbonate disks. The resulting cylinder was irradiated along directions orthogonal to the cylinder axis. Irradiation from multiple angles was obtained by rotating the cylinder around its axis, with the purpose of enhancing, as in actual radiotherapy, the dose deposition on a volume at a given depth of about 5 cm. This depth value is already of interest for some kind of tumors^65^, although it is clear that most of the clinical cases require the enhancement of dose at deeper regions. This is usually accomplished using more complex irradiation configurations; with this respect, our kind of experiment makes up a necessary building block for such developments. It is worth mentioning here that the typical values of the maximum electron energy envisaged for treatments of deep tumors are well within the spectral range observed in our experiment^48^.

Gafchromic films were used as detectors, sandwiched between neighbouring disks; irradiation thus occurred with the e-beam parallel to the disk surface, enabling in this way the high resolution sampling needed for a 3D dose deposition reconstruction with the required accuracy.

In the second case (IM), the phantom consisted of thin (2 mm thickness) polycarbonate slabs piled up to make a parallelepiped shape. Irradiation occurred with the e-beam propagating orthogonally to its smallest face. The so-called Intensity Modulation (IM), which basically aims at tailoring the dose deposition pattern in the transverse direction, was mimicked by translating the phantom in different positions along a plane orthogonal to the beam direction and by acquiring a different number of LPA bunches on each position.

**Figure 4:**
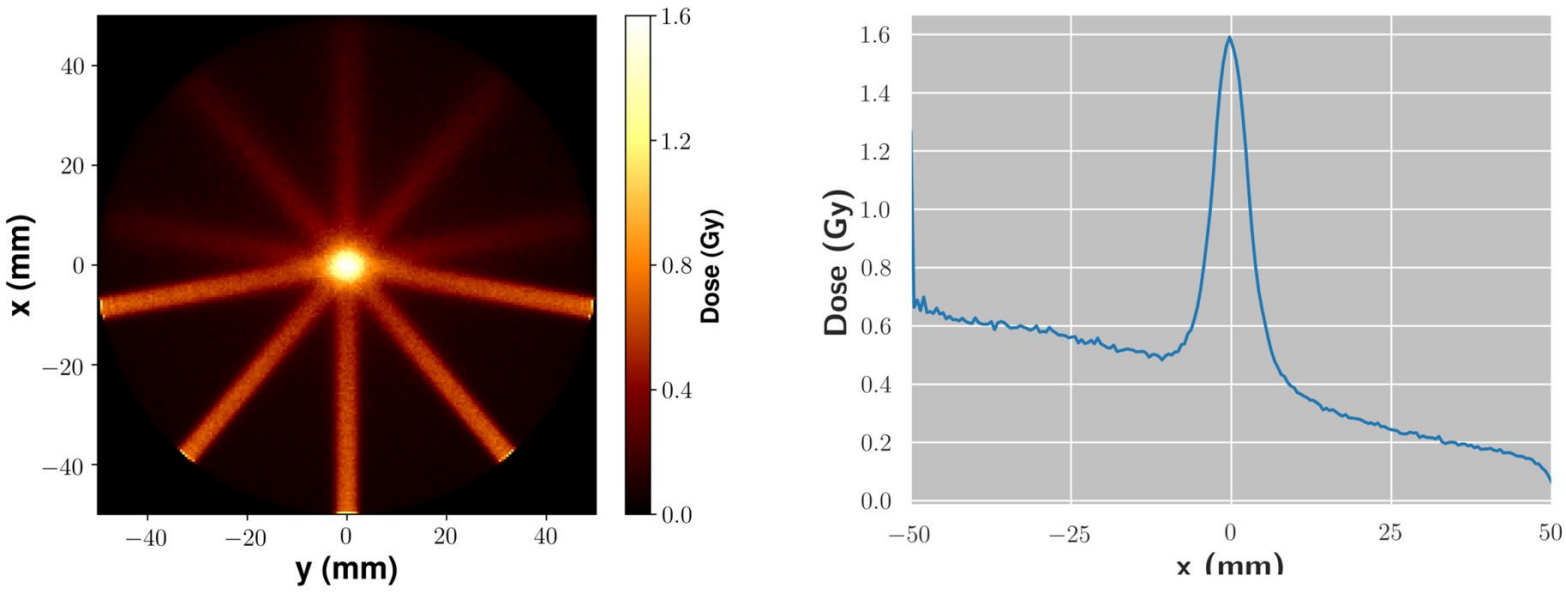
Dose distribution as obtained by Monte Carlo simulations in the MF irradiation case: left. 2D slice along the *z* direction, taken at the *z* position corresponding to the e-beam axis. right. Lineout along the *x* direction, averaged over a 1 mm distance in both the *y* and *z* directions.

#### Multi-field irradiation

Figure 3 shows the results of the MF irradiation using five fields (i.e., angles) at $40^\circ$ to each other and 40 LPA bunches for each field. Figure 3a provides a 3D rendering of the dose deposition pattern, by showing some isodose surfaces (see caption for details). Figure 3b shows a 2D slice along the *z* direction (that is, along the direction of the cylinder axis), at the position of the e-beam. The peak of the dose sitting out of the phantom center is due to a slight misalignment of the phantom (cylinder axis) with respect to the beam direction. Finally, Fig. 3c shows a slice in the *y* direction, taken at the position of the central field (that is, at $y=0$). We preliminarily observe that non negligible differencies in the dose deposition profile do appear among the different fields, in particular at small depths. This is likely due to corresponding fluctuations of the bunch charge at low energy ($\lesssim$  50 MeV), demanding a careful management of such spectral components for a real clinical application; we will briefly discuss this point below. It is clear from Fig. 3 that a dose enhancement occurs on a small volume at the overlapping region of the different fields, with a size comparable to the e-beamlet transverse size. In particular, the maximum dose within this volume is a factor $\sim 2.5$ of the dose at the entrance and up to a factor $\sim$ 3–4 with respect to the dose a few mm apart from the “target” volume itself (and a factor up to $\sim 16$ with respect to the dose toward the exit of the phantom). We mention here that the ratio of the dose in the “target volume” to that in the neighbouring region or in regions at higher depths strongly depends, ultimately, on the actual irradiation configuration, which can be optimized to deliver improved patterns. Our results confirm the relatively low dose lateral spread of VHEE and their capabilities for precise dose tailoring, as mentioned in the Introduction. This behaviour was confirmed by GEANT4 simulations, whose results are shown in Fig. 4, where a pretty clear pattern of the dose deposition from each field over the entire phantom diameter, as well as a clear enhancement of dose at the overlapping volume, are visible.

**Figure 5:**
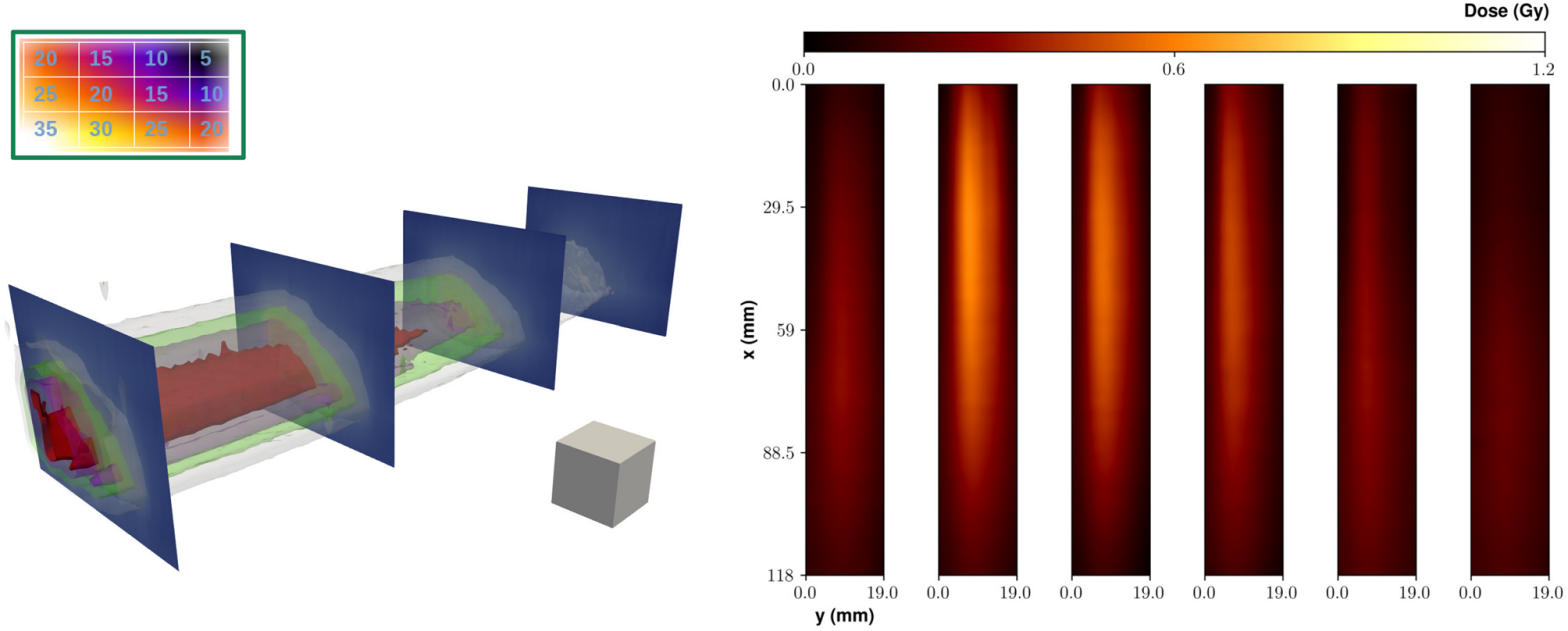
Top left inset: Number of shots used on each point of a $3\times 4$ array of positions; the distance between neighbour positions was 3 mm. Left: Isodose surfaces for selected dose values retrieved in the IM experiment. Red: 1.0 Gy; pink: 0.8 Gy; green: 0.6 Gy; grey: 0.4 Gy. As a visual aid, slices at different depths (20, 40 and 60 mm) are also shown. A small cube with 5 mm size is also shown as a spatial scale reference. Right: 2D maps of the experimental dose distribution retrieved on each gafchromic film in the IM experiment. The distance between adjacent layers was 2 mm.

#### Intensity modulation

The results of the IM experiment are shown in Fig. 5. In this case, we irradiated the parallelepiped shaped phantom at different positions separated by 3 mm on a $3\times 4$ matrix. We accumulated a different number of bunches (laser shots) on each position so as to create a transverse gradient of dose according to a predefined pattern. The number of bunches on each point is reported in the upper-left inset of Fig. 5. On the right of Fig. 5, the 2D maps of the dose values measured on each gafchromic film are shown. These maps clearly show the dose gradient along the *y* direction (on the same gafchromic film) and the *z* direction (different films). Starting with these data, a 3D dose distribution map was reconstructed. Figure 5*left* presents some isodose surfaces within the phantom; as a visual aid, some slices at selected planes at different depths are also shown. As it is evident, the dose deposition profile follows, to a good extent, the initial (predefined) transverse gradient, addressing the possibility of a localized dose deposition offered by this LPA based approach to irradiation with VHEE.

**Figure 6:**
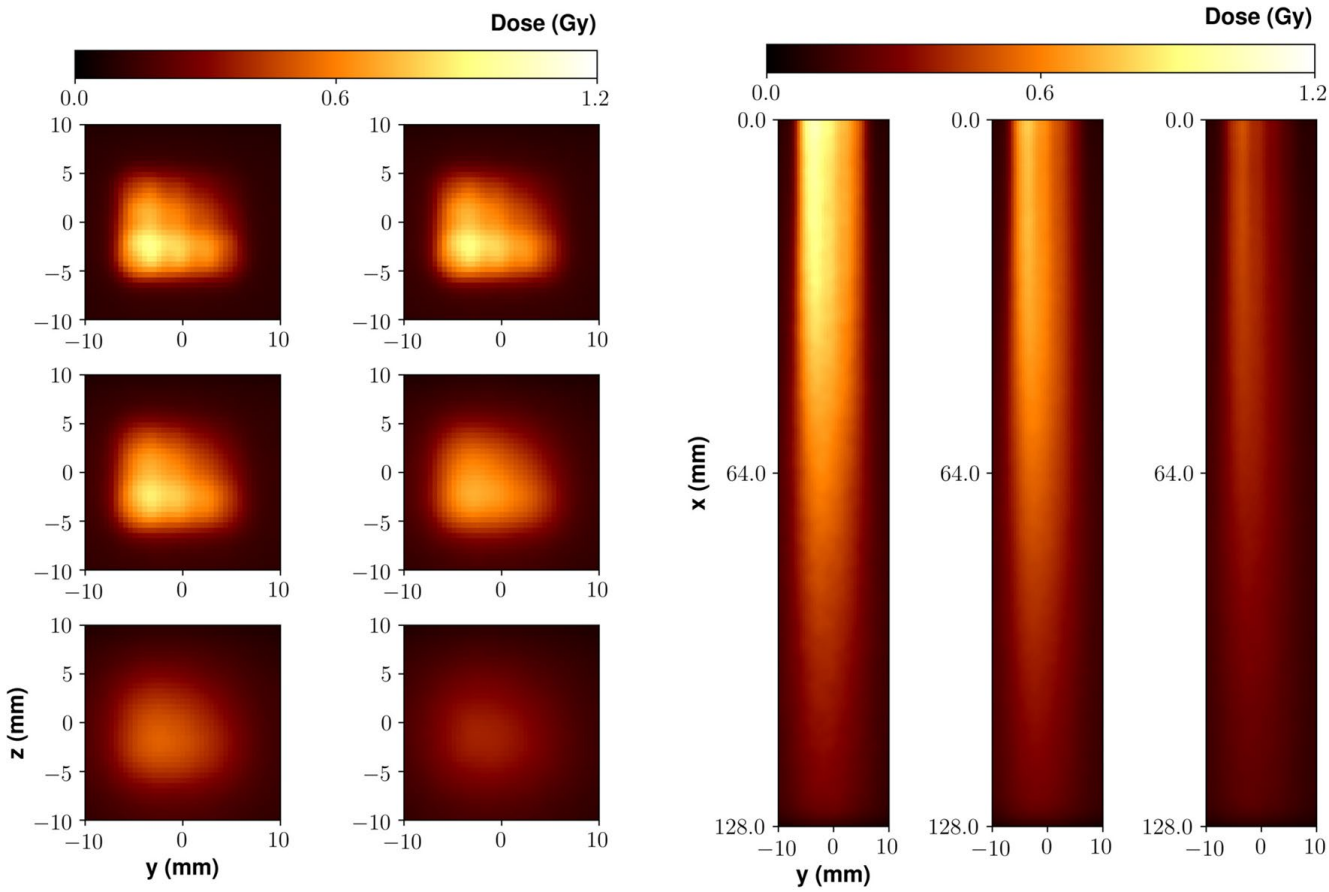
Dose distribution for the IM experiment as retrieved by Monte Carlo simulations. Left: Slices along the *x* direction. Left-to-right, top-to-bottom: $x=10.0$ mm, $x=20.0$ mm, $x=30.0$ mm, $x=50.0$ mm, $x=75.0$ mm, $x=100.0$ mm. Right: Slices along the *z* direction: left-to-right: $z = 0.0$ mm, $z = +3.0$ mm, $z = +6.0$ mm (position $z=0.0$ mm corresponds to the lowest row in the top left inset of Fig. 5).

As in the case of the MF experiment, we carried out Monte Carlo simulations to model our experimental observations. Results are shown in Fig. 6, which are in a good agreement with the experimental dose pattern. Looking at these results, some small scale spatial features appear, which are only weakly visible on the experimental data, possibly due to the limited sampling along *z* in this latter case; these features are due to the overlapping of adjacent beamlets, and are also a clear confirmation of the good dose deposition confinement properties of such high energy beams.

## Discussion and conclusions

The results presented in this paper demonstrate the feasibility of experimental studies related to electron radiotherapy of deep seated tumors with existing LPA electron sources. While several theoretical and numerical works dealing with this kind of treatment, including the development of Treatment Plannings, have been carried out so far, experimental works only focused on basic issues concerning VHEE beam dosimetry and radiobiology. As detailed in the Introduction, the appealing features in terms of dose deposition patterns exhibited by VHEE, actually provide solid motivations for further investigations in the field. The PDD, the MF and IM results in water phantoms shown here using an LPA accelerator confirm that a significant dose relevant for pre-clinical studies can already be delivered to deep seated targets, limited here only by experimental contraints in the specific LPA set up used.

As it is clear from the literature in the field, radiotherapy with VHEE is currently attracting a growing interest. Beside the increasing suggestions that VHEE may actually show comparable (or even better) performances to photon radiotherapy for certain kind of tumors, in terms, for instance, of dose volume histograms, evidence have recently emerged related to the potential offered by magnetic focusing of VHEE beams, in contrast to the case of photons. For instance, in a recent paper on magnetic focusing of VHEE, a noticeable improvement in the PDD curve was found in simulations^66^. Furthermore, the possibilities offered by steering the electron beams by (fast) magnetic scanning, to reduce issues related to physiological motion, is also considered an appealing feature^67^; this is in contrast to the relatively slow adjustement carried out in multileaf collimators.

We also mention here the recent boost of interest that direct VHEE radiotherapy has received as a consequence of the emergence of the so-called FLASH therapy paradigm^68^. The basic requirement for a FLASH treatment is a very high dose rate (exceeding $\sim$ 40 Gy/s) over a single (unfractionated) irradiation. Due to the very low efficiency of the electron-to-photon conversion of the *Bremsstrahlung* process used in the radiotherapy devices, delivery of this dose rate via high energy photons is currently out of reach and, anyway, extremely challenging. First attempts have recently been reported to get the required dose rates from ad hoc modified conventional machines^69^. However, it is envisaged that VHEE beams may be needed for deep tumors to be treated, at least in a first stage. In view of these circumstances, LPA sources are considered among the most promising candidates for future FLASH machines^70^.

Finally, we want to stress that several open issues still remain, calling for further studies before full “clinical quality” irradiation with LPA beams can be achieved. As it emerged from our experiment, for instance, the shot-to-shot reproducibility of the electron bunch parameters need to be improved. As an example, in order to comply with the requirements on the dose reproducibility for clinical treatments, bunch charge fluctuations should be limited within a few percent (see for instance^71^); this value was estimated to be about one order of magnitude higher in our experiment (see “Methods” section). Moreover, the spectral features of the bunch clearly affect the longitudinal and transverse behaviour of dose deposition. Fluctuations in the spectrum (see Supplementary Materials), for instance, may lead to imbalances among the dose patterns of the different fields in the MF irradiation, as visible from the above results. As it was anticipated above, this work was focused primarily on demonstrating control on deep dose deposition. In contrast, no special effort was dedicated here to the control of the electron energy spectrum for optimized PDD. Clearly, in order to keep dose deposition localized (along the longitudinal direction) and to enable a better dose spatial tailoring, electron bunches with enhanced spectral properties with respect to the ones used here are required. It is worth noticing here however, that most methods developed so far to enhance the “beam quality” of LPA electron bunches to reduce energy spread^72–74^, usually results in an excessive reduction, as high as one order of magnitude, of the bunch charge and dose-per-pulse. In this respect, the spectral requirements for clinical applications of VHEE will have to be carefully evaluated. Magnetic selection devices may offer the most effective solution to remove unwanted spectral components (for instance at low energy), that usually characterize high charge LPA bunches. Magnetic selection will also introduce the required degrees of freedom to adapt the spectrum and the dose deposition to the specific TPS, allowing a stable operation of the LPA source. Investigation of these issue will be the subject of future works. With this respect, we mention that very encouraging results have been reported very recently^75^ concerning the ability of LPA sources to sustain long operation times with remarkably high bunch properties stability. Furthermore, research on advanced online dosimetric devices is currently ongoing, which is seen as a fundamental step toward a clinical translation^52,62,76^.

To summarize, in our work we focused on the experimental investigation of the practical feasibility of complex irradiation configurations with LPA VHEE beams. Our study aimed at tailoring dose deposition to match the requirements for treatments of deep tumors, thus providing a testbed for future experiments. Our LPA source was optimized so as to provide a “pencil beam” suitable to target millimeter size volumes at depths up to $\sim$ 10 cm. Typical dose values up to a few cGy per laser shot were obtained, while the capability to build up doses of up to a few Gy over few mm size volumes at few centimeters depth, using $\sim 100$ bunches (laser shots) with multi-field irradiation, has been demonstrated for the first time. Furthermore, the possibility of tailoring the transverse dose profile with mm resolution, similar to what is usually accomplished with the so-called intensity modulation, has been successfully explored. While work is still needed to establish parameters required for clinical translation, we believe that our study clearly demonstrates the readiness of a platform for pre-clinical in vitro and in vivo studies based on LPA.

## Methods

### Laser-driven electron acceleration stage

The electron bunches were produced by Laser Wakefield Acceleration using the $220\,\mathrm {TW}$ laser system at the Intense Laser Irradiation Laboratory at CNR-INO. The beam, with $<30$ fs duration pulses, was focused using an $f/\sim 22$ Off-Axis Parabola down to a spot with waist $w\simeq 30\,\upmu$m. The energy in the focal spot was estimated to be $\sim 3$ J, providing an intensity $I_{max}\simeq 6\times 10^{18}\,{\mathrm{W}}/{\mathrm{cm}}^2$. The gas-jet was produced using a rectangular nozzle $1.2\,\mathrm {mm}$ long (along the laser propagation direction). A gas mixture He–N$_2$ (2.5% N$_2$) was used as target, at a backing pressure of $8\,\mathrm {bar}$. The plasma electron density profile had been previously characterized via optical interferometry^77^. Plasma parameters were diagnosed using Thomson Scattering imaging and side imaging (see Fig. 1). The laser-plasma interaction and the acceleration process were simulated using the Particle-In-Cell code FBPIC^78^. We refer to the Supplementary Materials for a discussion about that.

### Electron bunch diagnostics and collimation

The electron spectrum was measured using a magnetic spectrometer based on a magnetic dipole with maximum field $B\simeq 1.23$ T, a length (along the incoming electron beam direction) of 50.8 mm and a gap of 5 mm. The real magnetic field map, used for the tracing of the electron trajectories, was retrieved by comparison of field experimental measurements with the predictions of the software RADIA^79^. A LANEX screen was used as a detector, whose scintillation emission was imaged out using an EM-CCD camera. The LANEX screen (and the associated imaging optical tools) was placed outside of the vacuum chamber. A $50\,\upmu$m kapton window was used as vacuum-air interface for the electrons. The divergence of the e-beam was measured by removing the magnetic dipole and letting the beam directly impinge on the LANEX screen. The estimated value was $\simeq 14$ m rad r.m.s. in the acceleration regime selected for the experiment. The shot-by-shot fluctuations of the charge were also studied with the same configuration (that is, by removing the magnetic dipole), and estimated to be of about $15\%$ r.m.s. (measured over the region selected by the collimator). We observe that this only provides a relative measurement, although nearly independent of the electron energy^80^.

A collimator was used to make an angular selection on the beam and get the required pencil beamlets. The collimator was designed, with the aid of Monte Carlo simulations (see below) so as to reduce the X/$\gamma$-ray photons produced via *Bremsstrahlung* in the collimator structure and reaching the phantoms. The expected photon contribution to the total dose was predicted to be $>4$ orders of magnitude smaller that that from VHEEs (see Supplementary Materials). The collimator consisted of a sandwich-like structure made up by two plastic (PVC) disks with 80 mm diameter and 20 mm thickness (on the front and the rear side), and a 7.5 mm thick Pb disk in between. A central hole with 4.5 mm diameter was drilled on the three disks. Inside this hole, a plastic (teflon) cylinder was inserted along the whole thickness (47.5 mm), with a central aperture with 2 mm diameter. A $10\,\upmu$m thick mylar foil with a thin aluminum layer was placed in front of the collimator to prevent laser light from reaching the detectors.

### Electron beam dosimetry

Dosimetric study was carried out using two different phantoms, namely a stack of solid water (PLASTIC WATER, CIRS Inc.) sheets with increasing thickness (from 1 to $50\,\mathrm {mm}$) and a water filled tank. The tank had external size of $298\times 108\times 108\,\mathrm {mm^3}$ (refer to Fig. 1 for the orientation). In both cases, type EBT3 gafchromic films were used as a detector^81^. The film batches were calibrated in dose using a conventional radiotherapy machine at the S. Chiara Hospital in Pisa. EBT3 response was calibrated for both incoming electrons propagating along the normal to their surfaces and along the surface; the first configuration was used for the dosimetric studies, the second for the MF and IM irradiation studies.

### Monte Carlo simulations

Monte Carlo codes were developed in order to simulate the transport and interactions of the e-beam in all the experimental conditions reported. The codes were based on the GEANT4 toolkit^82,83^. The G4EmPenelopePhysics physics list constructor was used, with X-ray fluorescence also included; cut range was fixed to $10\,\mathrm {\mu m}$ in all volumes. All the elements downstream of the gas-jet nozzle (that is, collimator, vacuum chamber flange with kapton window, phantoms) were considered in the simulations, including their detailed structure. The primary electrons were generated, using an acceptance-rejection sampling method, according to the distribution function retrieved from the experimental data. Energy deposition in the phantom was sampled with a typical voxel size of 0.5 mm. For each run, $5\times 10^7$ primary electrons were used. Total running time for each run was of the order of 10 hours on a desktop PC equipped with an AMD FX6350 CPU.

## Supplementary information

Supplementary Information.

## Data Availability

The dataset generated and/or analysed during this study, as well as the numerical codes developed, are available from the corresponding authors on reasonable request.
