# Supplementary material for: Toward an effective use of laser-driven very high energy electrons for radiotherapy: Feasibility assessment of multi-field and intensity modulation irradiation schemes
Source: Sci Rep. 2020 Oct 14;10:17307. doi: 10.1038/s41598-020-74256-w (PMC7560873; doi:10.1038/s41598-020-74256-w)
Supplement: Supplementary file 1 — Supplementary Information. [file 41598_2020_74256_MOESM1_ESM.pdf]

Toward an effective use of laser-driven very high energy electrons for radiotherapy: Feasibility assessment of multi-field and intensity modulation irradiation schemes Supplementary Materials

Luca Labate[1,], Daniele Palla[1], Daniele Panetta[2], Federico Avella[1], Federica Baffigi[1], Fernando Brandi[1], Fabio Di Martino[3], Lorenzo Fulgentini[1], Antonio Giulietti[1], Petra Köster[1], Davide Terzani[1,4], Paolo Tomassini[1], Claudio Traino[3], Leonida A. Gizzi[1,**]*

[1] Consiglio Nazionale delle Ricerche, Istituto Nazionale di Ottica, Pisa, Italy

[2] Consiglio Nazionale delle Ricerche, Istituto di Fisiologia Clinica, Pisa, Italy

[3] Unità Operativa di Fisica Sanitaria, Azienza Ospedaliero-Universitaria Pisana, Pisa, Italy

[4] Now at Lawrence Berkeley National Laboratory, LBL, Berkeley, CA, United States

[*] luca.labate@ino.cnr.it

[**] la.gizzi@ino.cnr.it

Characterization of the LWFA regime

Electron bunch experimental characterization

As said in the main text, the divergence and spectrum of the electron bunch were measured using the setup described in the Methods. The FWHM divergence (measured by removing from the e-beam path the collimator and the magnetic dipole) was measured to be of $\sim 14\,\mathrm{mrad}$. A spectrum, obtained upon averaging over 10 laser shots, is shown in Figure 1. Our spectrometer range had a lower bound of $\sim 50\,\mathrm{MeV}$. The experimental spectrum featured a high energy component peaking at $\sim 200\,\mathrm{MeV}$. A strong component was also present in the range $\sim 50 - 125\,\mathrm{MeV}$, whose origin was clarified by means of Particle-In-Cell (PIC) simulations, discussed in the following Subsection.

The PIC code

PIC simulations have been carried out to get a better undertsanding of the acceleration mechanism in our regime and check the main experimental bunch parameters against the simulated ones. Simulations were carried out using the FBPIC code [1] on a single 32GB NVIDIA Tesla V100 SXM2 GPU. FBPIC is a spectral, quasi-cylindrical PIC code based on an azimuthal Fourier decomposition; it does not suffer from numerical dispersive errors in the electromagnetic field

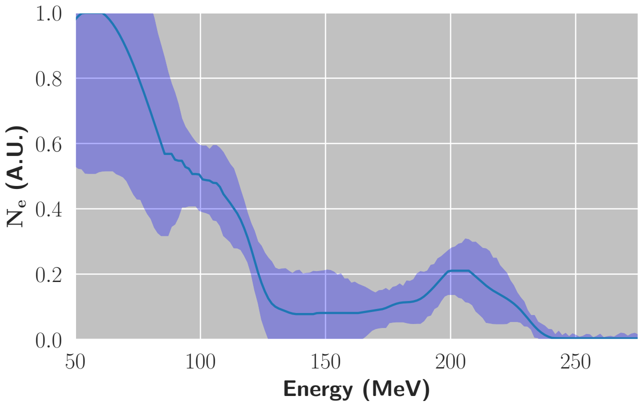

Figure 1: Experimental electron spectrum, averaged over 10 laser shots. The r.m.s. is shown as a coloured area (for the sake of a better readability, the upper extrema are not visible at energies $\lesssim 80\,\mathrm{MeV}$; the upper interval is however comparable to the bottom one in this region).

propagation. In order to take into account a linearly polarized laser pulse, the code was run using two azimuthal modes (see below).

Simulations

We approximated the laser driver as a bi-gaussian pulse, both in the longitudinal and transverse directions, with an energy $\mathcal{E} = 3\mathrm{J}$, a waist $w_0 = 30\mu\mathrm{m}$ and a longitudinal duration $\tau_{fwhm} = 30\mathrm{fs}$, corresponding to a laser strength parameter of $a_0 \simeq 1.75$. Based on previous interferometric measurements, the density profile was modelled using an initial and final linear ramps, each with length $250\,\mu\mathrm{m}$, and a $900\,\mu\mathrm{m}$ long plateau, where the (peak) density of the neutral gas was $n_0 = 4\times10^{18}\,\mathrm{cm}^{-3}$. We modeled the laser-plasma interaction using the standard moving window technique, using a simulation box with $\sim 3w_0$ transverse size; in the longitudinal direction, the size was adjusted so as to fit both the laser pulse and the first trailing plasma oscillation. The single cells composing the electromagnetic lattice had longitudinal size $\Delta z = \lambda_0/32$ and radial size $\Delta r = \lambda_0/12$. The background plasma was described employing 16 computational particles per cell. As said in the text, the gas was a mixture of He and N_2, with 2.5% at. N_2 concentration.

At laser intensities of the order of the one at play in our experiment, we expect two concurrent processes to contribute to the electron bunch formation, namely the electron self (or *bubble*) injection and the ionization injection. Some parametric scans have been carried out to investigate whether one of the two was dominant in the bunch generation to ensure an optimized choice of the numerical parameters in use. In particular, from previous experiences, we know that to provide a decent modelling of the self injection mechanism, FBPIC requires at least 3 azimuthal modes. The ionization process is instead strongly dependent on the longitudinal cell size, since

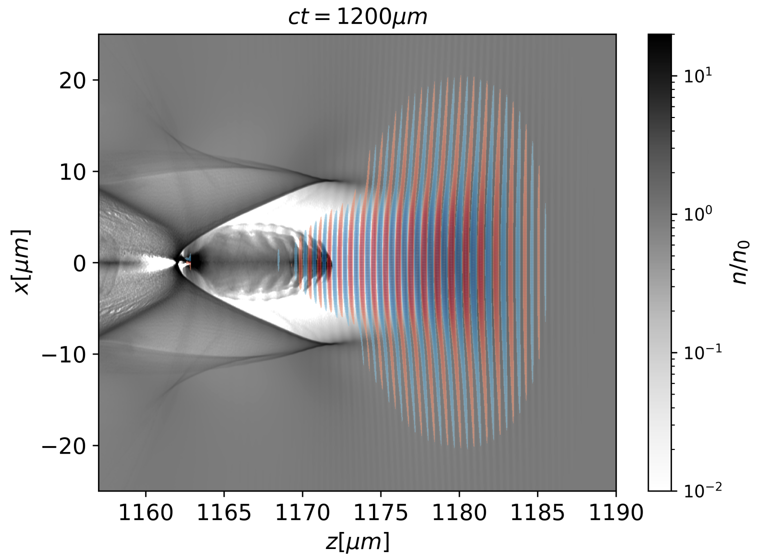

Figure 2: Density log map of the background plasma after a laser propagation of $ct = 1200\mu\mathrm{m}$. An injected particle bunch is visible in the center of the plasma bubble. The oscillating laser field is shown and the self-focusing effect is clear on the rear of the pulse.

the laser oscillation must be well resolved. We thus initially performed a numerical scan at a reduced longitudinal resolution using 3 modes. This scan showed a negligible contribution of the self injection mechanism, so that we could run further simulations with an increased longitudinal resolution with 2 azimuthal modes. FBPIC is equipped with an ionization module based on the well-established ADK model, that randomly extract an electron from every nitrogen background atom.

Results

In Figure 2, we show the density map of the background plasma when the pulse is located around the center of the gas jet. We can see the laser pulse focusing effect giving rise to the characteristic plasma bubble accelerating a very long injected bunch, that extends from $z \sim 1163\mu\mathrm{m}$ to $z \sim 1173\mu\mathrm{m}$. Figure 3 shows the electron Lorentz gamma factor overlapped to the longitudinal accelerating electric field.

We show in Figure 4 the electron spectrum as provided by the PIC simulation at the end of the plasma; for the sake of a comparison with the epxerimental spectrum, a selection on the transverse momentum was performed, by retaining only particles entering our collimator (see main text). Beside a very low energy component ($E \lesssim 30\,\mathrm{MeV}$), two broad peaks can be

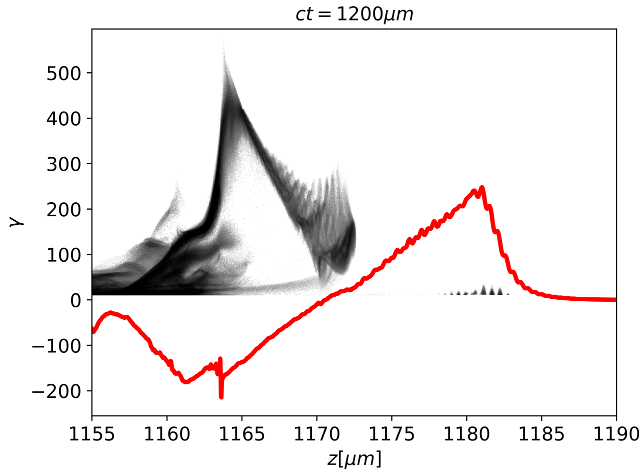

Figure 3: Particles gamma as a function of the longitudinal position. Red lines represents a centerline of the accelerating field normalized to be visible in the plot. The front of the particle bunch is dephased and does not accelerate anymore. The center of the bunch is the most energetic part, while the rear, carrying the greatest part of the charge, produces a strong beam loading effect.

identified, located at relatively low energy ($\simeq 50\,\mathrm{MeV}$) and at $\simeq 200\,\mathrm{MeV}$. By following the phase space distribution in time we found out that the peculiar double-peaked energy spectrum is due to the underlying injection mechanism. Indeed, at the first stages of the acceleration dynamics, the laser pulse extracts some (newborn) electrons, following the ionization from N^{5+} to N^{6+} that get trapped in the wakefield and start to accelerate. While the laser pulse self-focuses, the wakefield amplitude and wavelength increase, and the first extracted particles find themselves in a region near the zero of the accelerating field. On the other hand, particles extracted at a later time start to experience the highest accelerating field and therefore reach higher energies.

However, since the ionization mechanism does not stop until late in the process, electrons are continuously injected in the wakefield, increasing the beam loading effect and therefore slowing down their acceleration. The tail of the injected bunch contains a remarkable part of its charge, highlighted in Figure 5, which forms the low energy peak in the spectrum.

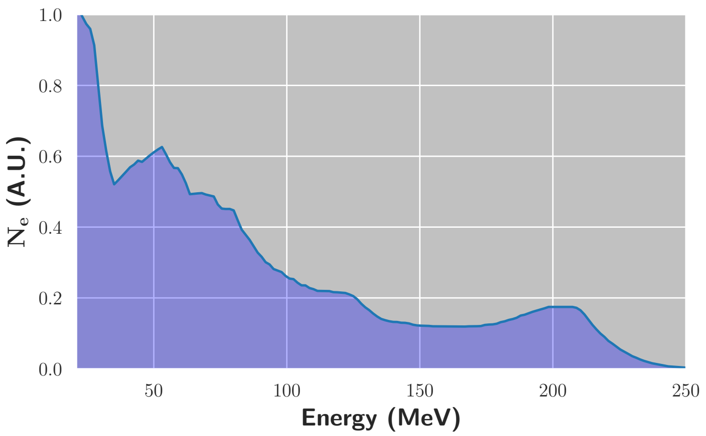

Figure 4: Electron spectrum at the end of the acceleration, taking into account only electrons entering our collimator (see main text).

Monte Carlo simulation of the dose contamination by photons

Bremsstrahlung (as well as fluorescence) photons are expected to be emitted in our experimental setup as a result of the interaction of the electrons with the structures of the collimator as well as of the flange/vacuum window. The contribution to the dose due to the photons was estimated using the Monte Carlo (GEANT4 based) simulations (see Methods in the main text). Figure 6 shows a 2D map of the dose in the water phantom due to the photon only (bottom plot); for the sake of comparison, the total dose in also shown in the top plot (this is the same plot as the one reported in the main text). As it emerges, the ”dose contamination” from photons is negligible.

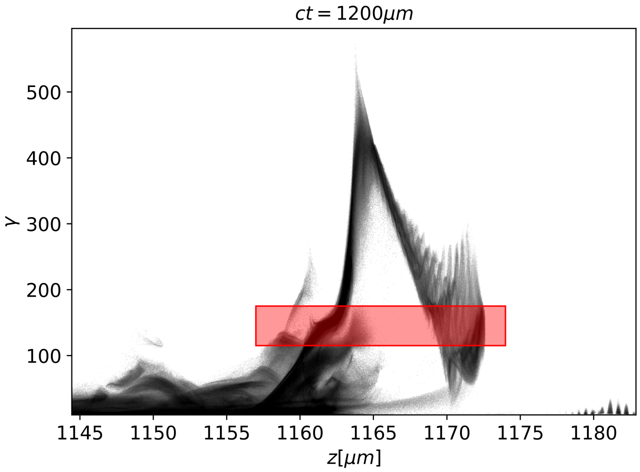

Figure 5: Same plot as in Fig.3, where particles forming the low energy peak in the spectrum have been highlighted by a red rectangle.

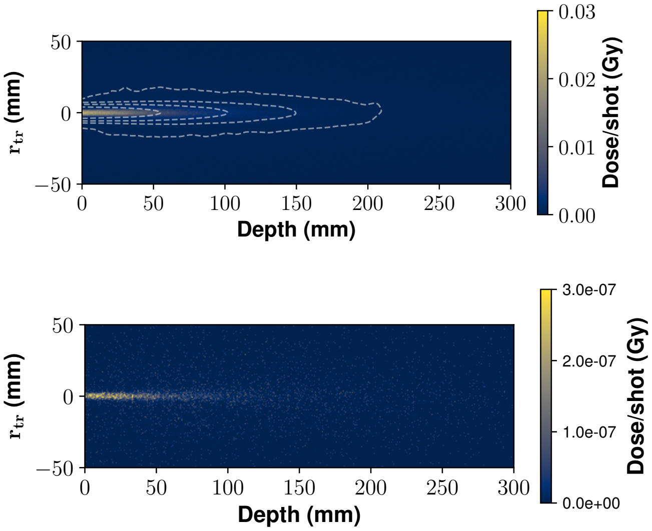

Figure 6: *Bottom:* 2D map of the dose deposited in the water phantom by the photons produced as a result of the interaction of the primary electrons with the collimator and flange/window structures. For the sake of an easier comparison, the total dose (i.e., dose from all the kind of particles) is also shown on the top.

Bibliography

[1] R. Lehe *et al.*, *Comput. Phys. Commun.* **203**, 66-82 (2016)
